# Working Memory Replay Prioritizes Weakly Attended Events

**DOI:** 10.1523/ENEURO.0171-17.2017

**Published:** 2017-08-16

**Authors:** Anna Jafarpour, Will Penny, Gareth Barnes, Robert T. Knight, Emrah Duzel

**Affiliations:** 1 Department of Psychology, University of California, Berkeley, California 94720; 2 Helen Wills Neuroscience Institute, University of California, Berkeley, California 94720; 3 Wellcome Trust Centre for Neuroimaging at University College London, London WC1N 3BG, United Kingdom; 4 Institute of Cognitive Neuroscience, London WC1N 3AR, United Kingdom; 5 German Centre for Neurodegenerative Diseases (DZNE), 39120 Magdeburg, Germany; 6 Institute of Cognitive Neurology and Dementia Research, Otto-Von-Guericke, University of Magdeburg, 39120 Magdeburg, Germany

**Keywords:** decoding, magnetoencephalography, maintenance, working memory

## Abstract

One view of working memory posits that maintaining a series of events requires their sequential and equal mnemonic replay. Another view is that the content of working memory maintenance is prioritized by attention. We decoded the dynamics for retaining a sequence of items using magnetoencephalography, wherein participants encoded sequences of three stimuli depicting a face, a manufactured object, or a natural item and maintained them in working memory for 5000 ms. Memory for sequence position and stimulus details were probed at the end of the maintenance period. Decoding of brain activity revealed that one of the three stimuli dominated maintenance independent of its sequence position or category; and memory was enhanced for the selectively replayed stimulus. Analysis of event-related responses during the encoding of the sequence showed that the selectively replayed stimuli were determined by the degree of attention at encoding. The selectively replayed stimuli had the weakest initial encoding indexed by weaker visual attention signals at encoding. These findings do not rule out sequential mnemonic replay but reveal that attention influences the content of working memory maintenance by prioritizing replay of weakly encoded events. We propose that the prioritization of weakly encoded stimuli protects them from interference during the maintenance period, whereas the more strongly encoded stimuli can be retrieved from long-term memory at the end of the delay period.

## Significance Statement

Here we show how information of a sequence of events is prioritized in the working memory maintenance buffer in humans. Participants retained three consecutive visual stimuli, and we decoded the content of working memory maintenance using multivariate pattern classification and magnetoencephalography. We observed that the least attended events during encoding dominated the content of working memory immediately following off-line retention. In essence, the brain selectively and intelligently amplifies the least encoded memory item to maximize recall fidelity, instead of equally rehearsing the whole sequence. Our findings shift the functional role of working memory from a faculty that “works with memory” to one that “works for memory” by actively selecting which encoded items need to be enhanced to be remembered.

## Introduction

Working memory is conceptualized as a mechanism to actively maintain and manipulate information (Baddeley, 1992). It is considered to consist of multiple layers, including long-term memory and a maintenance buffer , which is also known as the focus of attention during maintenance (Oberauer, 2002; Baddeley, 2010) that interacts with long-term memory. Working memory maintenance is associated with a reactivation of information in nonhuman primates (Miller et al., 1993; Lee et al., 2005; Woloszyn and Sheinberg, 2009) and in humans (Lepsien and Nobre, 2007; Harrison and Tong, 2009; Fuentemilla et al., 2010). Here we investigated the representational content of maintaining a sequence of multiple stimuli in working memory. To decode representational content, we used multivariate pattern analysis of magnetoencephalography (MEG) recordings (Jafarpour et al., 2013; Cichy et al., 2014).

We addressed two hypotheses. The first hypothesis was that stimuli are maintained in a circular and repetitive structure. This hypothesis was motivated by the temporal coding model of working memory maintenance, which proposes that the replay mechanism conserves the temporal order in which stimuli were encountered (Lisman, 2010; Jensen et al., 2014). Thus, the sequence of 1-2-3 circularly rehearses as 1-2-3-1-2-3-1-2-3-etc. Such a dynamic has been reported in the medial temporal lobe of rodents (Jensen and Lisman, 1996) and in the nonhuman primate prefrontal cortex (Siegel et al., 2009). Support for the temporal coding model also comes from a recent human MEG study (Heusser et al., 2016). In that study, fitting the temporal coding model to whole-brain the MEG data source localized evidence for the model in the human hippocampus (Heusser et al., 2016). However, the trial-by-trial activity of the prefrontal cortex of a nonhuman primate supports a dynamic coding model of working memory, rather than the temporal coding model (Lundqvist et al., 2016). The dynamic coding model suggests that items are maintained in an “activity silent state” and that replay is guided by attention (Stokes, 2015; Myers et al., 2017). Attention at encoding could thus prioritize the content of working memory such that working memory maintenance is dominated by a selected stimulus rather than the full to-be-memorized sequence. For instance, it would be more resource effective to prioritize the less privileged stimuli at encoding to be replayed in working memory (Zokaei et al., 2014; Stokes, 2015; Rose et al., 2016).

**Figure 1.**
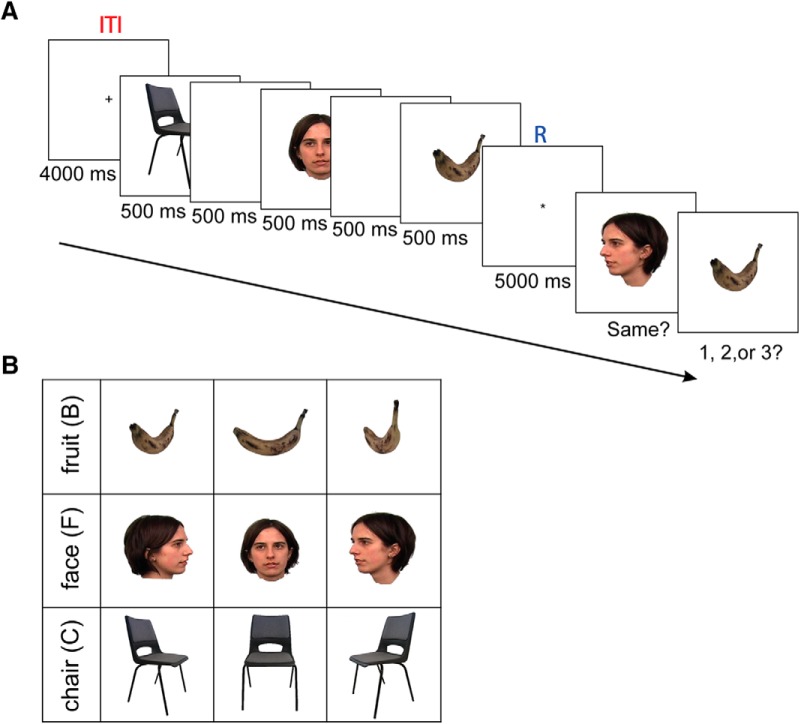
Working memory experimental paradigm. ***A***, Three stimuli were presented sequentially, each for 500 ms and with a 500 ms gap between them. There was a 5000 ms retention period in between the presentation of the third stimulus and memory probe tests. The memory probe tests entailed a “same” or “different” judgment and a temporal order decision. A 4000 ms intertrial interval preceded the next trial. The R period is shown in blue, and the ITI is shown in red. ***B***, The stimuli were used in this experiment as follows a B, an F, and a C from three points of view, 60° to the left, front on, 60° to the right.

Here we used the whole-brain MEG data to decode the content of working memory. Our experiment was a modified version of the Sternberg task, where a sequence of three visual stimuli had to be retained. Objects from three distinct visual categories [faces (Fs), manufactured objects, and natural items] were presented successively (the stimulus set contained samples of the same items from different perspectives; Fig. 1*B*) followed by a 5000 ms delay period. After the delay, a probe queried stimulus identity (detail test) and a second probe queried the sequence of the three items (first-, second-, or third-order test; Fig. 1).

**Figure 2.**
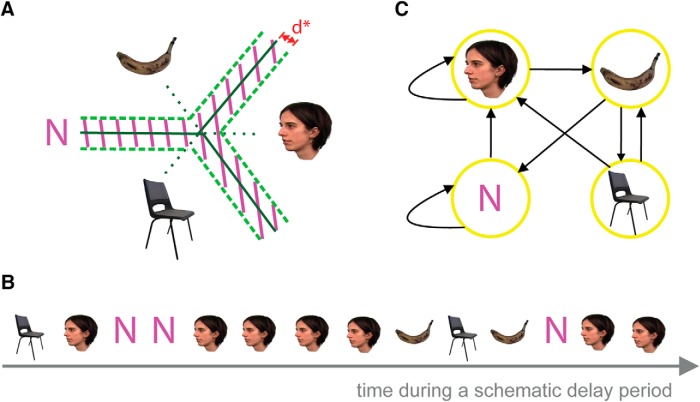
Schema of the multivariate pattern analysis using SVM. ***A***, The state of neural activity during delay periods (R period or ITI) was decoded at each time bin, using three pairwise classifiers. A conservative threshold of d* (depicted in red) was used to reject representations that were close to the boundary and categorize them as N (the shaded area). ***B***, A schematic example of decoded states during a delay period. ***C***, The discrete time Markov chain model of state transition extracted from the schematic sequence in ***B***.

Pattern classifiers were trained on categorical representations of visual stimuli in brief time bins (20 ms) during encoding (Carlson et al., 2013; Jafarpour et al., 2014). The classifiers labeled the ongoing signal during retention (R) and intertrial interval (ITI) periods for control. According to the output of the classifiers [face, banana (B), chair (C), or “none” (N) for no replay], a Markov chain matrix of transitions between replayed stimuli and none was constructed (Fig. 2). With three stimuli, we could test for the direction of replay (i.e. 1-2-3 vs 3-2-1). A Markov chain matrix of transitions quantified the directional replay of sequences. The probability of transition from state 1-2, 2-3, and 3-1 would be higher than the probability of transition from state 1-3, 3-2, and 2-1 if there is a forward replay and the reverse pattern would be observed for backward replay.

A support vector machine algorithm was used for decoding the (pairwise) categorical information at −20 to 500 ms from onset of the visual stimuli during encoding. Note that the categorical representation and item-specific representation overlaps in our case, because we used only one sample from a category in this study (Fig. 1*B*). We trained the classifiers on the amplitude of the broadband event-related single-trial MEG signals and tested using a cross-validation method during encoding. We applied the classifiers with best performances to decoding during the maintenance interval. To determine the degree of attention during encoding, we analyzed early event-related fields (ERFs) to each stimulus.

The sequential mnemonic replay hypothesis would predict decoding sequence information or at least an equal probability of decoding for all three encoded stimuli during maintenance. In contrast, an attentional prioritization account would predict that the degree of stimulus replay during the maintenance period would be dependent on the size of early ERFs at encoding.

## Materials and Methods

### Participants

Sixteen right-handed, healthy adults with normal or corrected-to-normal vision participated in this experiment [8 females; average age, 24 years (SD = 2)]. The MEG data from two participants were not included in the analysis, as their MEG signals were too noisy and were rejected as artifacts (for details, see below). All participants gave written informed consent and were compensated financially for their participation. The University of London Research Ethics Committee for Human-Based Research approved the study.

### Experimental design

We used a combination of a delay-match-to-sample and Sternberg tasks. The experiment consisted of six runs, and each run consisted of 27 trials. Participants had an optional 5 min break between runs. Each trial contained a sequential presentation of three stimuli, a retention period, and two probe tests. A trial started with a fixation (intertrial interval) period for 4000 ms. Then a random sequence of three stimuli appeared sequentially for 500 ms, with a 500 ms gap between stimuli. A 5000 ms retention period followed the presentation of the third item. Finally, a probe stimulus was presented to test for item memory (delay-match-to-sample), where subjects were required to select “same” if the exact stimulus (category and perspective) was shown in the sequence and “different” otherwise (the perspective was different). Randomly, in half of the trials, the correct answer was “same”. For the following question, subjects were required to answer “1, 2, or 3” according to the position of the probe in the sequence (Fig. 1*A*).

The stimuli were images from three visual categories for which previous multivariate decoding research indicated the following distinct spatial cortical representations (Kriegeskorte et al., 2008): a face, a fruit, and a manufactured object (Fig. 1*B*). Images were from three different perspectives (front-on, 60° to the left, and 60° to the right) shown upright on a white background, extending ∼6° of a horizontal and vertical visual angle (face images were downloaded from Faces stimulus images Tarrlab, Center for the Neural Basis of Cognition and Department of Psychology, Carnegie Mellon University, Pittsburgh, PA; http://www.tarrlab.org/). Subjects were familiarized with the stimuli outside the MEG scanner, and they also performed the experiment with feedback outside the scanner to ensure that they understood the experiment properly. There was no feedback given during the experiment inside the MEG scanner. In six runs each with 27 trials (altogether, there were 162 trials), we tested all possible sequential combinations of three stimuli. All of the possible combinations of three stimuli are 162 sequences: six combinations of sequences of three categorical stimuli, and three perspectives of each stimulus category (= 6 × 3 × 3 × 3). We presented the trials randomly, and each trial was seen once.

### MEG recordings and data preprocessing

MEG data were recorded with a 274-channel CTF Omega whole-head gradiometer system (VSM MedTech) with a 600 Hz sampling rate with an on-line bandpass filter from 0.1 to 200 Hz. The head position inside the system was tracked via head localizer coils attached to the nasion and 1cm anterior to the left and right preauricular points. Participants sat upright, and the stimuli were back-projected onto a screen 1 m in front of them.

MEG data were preprocessed using SPM12b (Wellcome Trust Center for Neuroimaging, London, www.fil.ion.ucl.ac.uk/spm) package and analyzed using MATLAB R2009b software (MathWorks). We filtered out the main noise (50 Hz) from a continuous signal using a fifth-order Butterworth filter. We cropped the MEG data during encoding to epochs from −100 to 500 ms from the stimuli onset. We discarded any epoch with field magnitudes >1.5e-11 tesla in any channel, because it contained artifacts. Two subjects had too many trials with such artifacts and were removed from further analysis.

### Decoding the category of visual stimuli during encoding

A support vector machine (SVM) with a linear kernel (Vapnik, 2000), implemented in MATLAB statistics software, was used to classify the signal elicited by the onset of the visual stimuli. Twenty-six classifiers were adopted at −20 to 500 ms from stimulus onset during encoding. The sampling rate of the signal was 600 Hz. The signal was windowed in time bins of 20 ms (13 time points in each time bin), centered at −10, 10, 30, 50, 70, 90, 110, 130, 150, 170, 190, 210, 230, 250, 270, 290, 310, 330, 350, 370, 390, 410, 430, 450, 470, and 490 ms. The single-trial input to the SVM classifiers was the broadband amplitude at each time point and each channel (13 × 274 = 3562 features) for every stimulus. The features were normalized before training, and the scale was used to normalize features in testing data. We used a two-tailed *t* test with a threshold of 0.05 for the feature reduction.

We trained three pairwise classifiers to decode the stimulus category at each time bin during encoding, irrespective of presentation order or perspective: face versus banana (F vs B), face versus chair (F vs C), and banana versus chair (B vs C). We identified the time bins with reliable category stimulus classification and trained the classifiers on 90% of randomly selected samples from each category and tested them on 10% of left-out samples from each category (i.e. 10-fold cross-validation). We selected an equal number of trials from each category for training and testing.

We examined the classification performance at the group level. To test the accuracy of each classifier against chance (i.e., 50%), we used a one-sample *t* test with a correction for multiple comparisons [familywise error (FWE)] using random field theory (RFT) implemented in SPM (Kilner et al., 2005; Litvak et al., 2011). As is standard in neuroimaging, we made inferences using a cluster-level threshold. The RFT procedure adjusts the *p* value statistics that are functions of the number of time points (classification repetition). Such adjustment is similar to a Bonferroni correction. However, a Bonferroni correction is suitable for datasets that are independent at each repetition (or data point). Here the data from adjacent time points is not independent, and RFT is more suitable for multiple comparison correction (Kilner et al., 2005; Jafarpour et al., 2014).

### Decoding the category of visual stimuli during delay periods

The most accurate classifiers from encoding were used to decode the replay during maintenance (the delay period between encoding and testing) and during the ITIs (Fig. 2). For the delay period, we restricted analysis to the 1000–4000 ms after the offset of the last stimulus in the sequence (150 time bins were tested) to exclude the event-related activity elicited by offset of the last stimulus. We selected the 3000 ms before the onset of the first stimulus in the sequence (again including 150 time bins) for testing the ITIs.

The outputs of the three pairwise classifiers were class labels (F, B, or C), and the distance between unknown activity and classification decision boundaries. We determined the decoded labels according to these outputs in two steps. First, we selected the class label (among three classifier outputs) that had the largest distance to decision boundaries. Second, we used a threshold to identify unknown activities that were too close to the classification boundaries. We rejected these decoded classes and labeled them as N.

A threshold was used to reject a percentage of classification outputs during the retention period. For example, if the classifier performance was reliable 80% of the time, we rejected 20% of the labels of the decoded time bins during retention. We applied the same conservative threshold on decoded output during ITIs. Following those steps, four possible labels resulted from the classifiers: F, B, C, or N (for rejected classifications; Fig. 2).

Two parameters were studied to quantify the differences in the decoding during the R period and the ITI on a trial-by-trial level. The first parameter was the number of consecutive time bins decoded as the same item (i.e., a decoding epoch). We compared the length of the decoded epoch between the R period and the ITI. We trusted that the decoded items were replayed only when the memory benefited from the decoding (see the analysis on the effect of active maintenance on behavioral responses).

The second parameter was the dynamics of replay extracted by the Markov chain. We treated the classifiers outcomes as a state and counted the number of visits to the states and transitions among them during the R period and the ITI. We then extracted the probabilities of transitions for each subject and compared them between the retention periods and intertrial intervals at the group level using a two-sided Wilcoxon rank sum test.

The directionality of replay was tested using a two-sided Wilcoxon rank sum test. We performed the following comparisons:

1. Probability of forward replay with the probability of backward replay. Assuming an independent probability of replay of each stimulus, the forward replay was the multiple of probability of transitions from the first stimulus to the second stimulus, from the second stimulus to the third stimulus, and from the third stimulus to the first stimulus. Backward replay was the multiple of probability of transitions from the third stimulus to the second stimulus, from the second stimulus to the first stimulus, and from the first stimulus to the third stimulus.
2. Probability of transitions from the first stimulus to the second stimulus with probability of transitions from the first stimulus to the third stimulus.
3. Probability of transitions from the second stimulus to the first stimulus with probability of transitions from the second stimulus to the third stimulus.
4. Probability of transitions from the third stimulus to the first stimulus with probability of transitions from the third stimulus to the second stimulus.

### Effect of active maintenance on behavioral responses

We applied a linear mixed-effects model to evaluate the effect of the length of a predominantly replayed epoch on the behavioral performance and response time across subjects. In each trial and for each probe (in both detail and order tests), we took the number of consecutive time bins that the probe was replayed as a fixed variable and the subject number as a random variable. The effect of replay on behavior was visualized by grouping the probes according to whether or not they replayed during retention period and if replayed, whether the replay epoch was long (>1100 ms; see Fig. 4) or short. We grouped the hit rate and response time accordingly. We studied the normalized behavioral performances and effect of active maintenance on behavior at the group level using ANOVA and paired-samples *t* test for *post hoc* tests, implemented in IBM SPSS Statistics version 23.

### Event-related field predicting predominant replay

We investigated whether ERFs during stimulus presentation predicted maintenance. During maintenance, one stimulus was predominantly replayed. We grouped an event-related response according to its replay during the retention period: if the stimulus was predominantly maintained during retention interval (PM) or not (non-PM). We studied the event-related field using SPM12b, and ERF signals were baseline corrected based on the averaged amplitude in the whole epoch and low-pass filtered at 20 Hz.

The significant effects were then source localized separately (an early effect peaked at 125 ms, and a later effect peaked at 278 ms). We cropped the signal to a 50–200 ms epoch to localize the first effect (115–135 ms), and cropped the signal to a 200–350 ms epoch to localize the latter effect (270–300 ms). ERFs were source localized using 8192 vertices over the cortical surface in MNI space, a single shell as a forward model, and multivariate sparse priors (Friston et al., 2008). The individual source-localized activity was then examined in a group-level statistical analysis (Henson et al., 2007).

## Results

### Pattern classifiers performance

**Figure 3.**
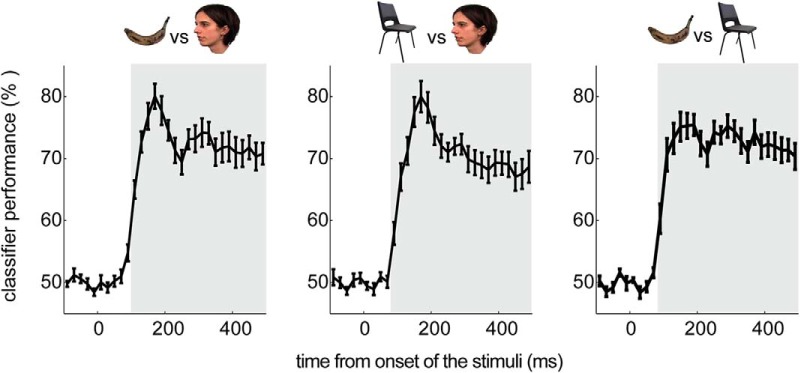
Multivariate classification of stimulus categories: cross-validation performance, these plots show the mean classification performance of 3 pairwise classifiers across the group: left, F vs B; middle, F vs C; and right, B vs C. The *x*-axis is the time from stimulus (0 ms), and the *y*-axis is the classification performance in percentage. The error bars show the SEM. The gray area indicates significant classification after correction for multiple comparisons.

We calculated the accuracies of three pairwise classifiers by averaging the classification accuracies over validation folds and paired categories. The results indicated that all classifiers performed at a better than chance level (50%) from ∼100 to 500 ms after the onset of the stimuli (from −10 to 490 ms tested time bins). F versus C classification performance was above chance from 90 ms after stimulus onset, with the highest performance of 80% at 170 ms (*t*_(13)_ = 14.76, *p* < 0.001, FWE corrected). The performance for the B versus C classifier was also significant from 90 ms, with the best performance of 75% at 190 ms (*t*_(13)_ = 14.61, FWE corrected *p* < 0.001). F versus B classification was significant from 110 ms, with 80% performance at 170 ms (*t*_(13)_ = 12.35, *p* < 0.001, FWE corrected; Fig. 3).

### Replay of one stimulus category dominates during retention

The 170 ms classifiers had the highest performance during encoding (the averaged cross-validated accuracy, over all three pairwise classifiers, was 78%). Thus, we selected the 170 ms classifiers for decoding within two time windows where maintenance may occur: the R interval itself and the ITI for control. Each period contained 151 time bins. Overall, we decoded ∼330,000 time bins.

**Figure 4.**
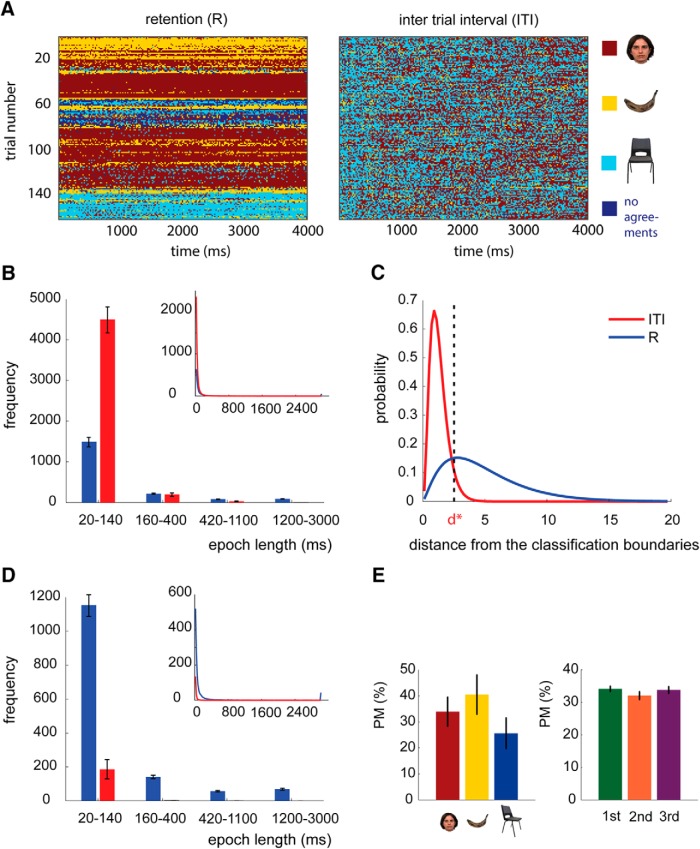
Decoding maintained categories in the delay period. ***A***, The plot shows a representative example (from one subject) of the decoded R period and ITI before thresholding. The *x*-axis is the decoded time bins, and the *y*-axis is the trial number. ***B***, The histogram of length of replay epochs during the R period (in blue) and during the ITI (in red) before threshold: the *x*-axis shows the epoch length. The top plot is the averaged epoch length from 20 to 3000 ms, and the bottom plot is the bar plot for bins of epoch lengths (20–140, 160–400, 420–1100, and 1200–3000 ms). Error bars show the SEM. The *x*-axis is length of the epoch of stimuli replay. ***C***, The probability distribution of distance from classification boundaries during the R period (blue) and ITI (red). d* shows the threshold for rejecting 22% of classification outputs during retention. This threshold rejected 94% of classification outputs during ITI. ***D***, The same histograms as in ***B*** but after applying the threshold. ***E***, The bar plots show the percentage of trials where the stimuli from the selected category (left plot) or order in the sequence (right plot) was predominantly maintained. There was no significant effect of category or the order of stimuli.

The distributions of assigned category labels to each time bin were different during the R period and ITI (Fig. 4). During the R period, the decoded adjacent time bins were most frequently from the same category (Fig. 4*A*, an example from a representative subject). We refer to these adjacent time bins with the same decoded categories as a replay “epoch”: it quantifies the length of time staying in the same state. The lengths of all epochs (multiple per a delay period) were then calculated, and the histogram of epoch lengths during the R period and ITI were compared in the four length bins: 20–140, 160–400, 420–1100, and 1200–3000 ms (note that a unit time bin was 20 ms). We observed shorter replay epochs during the ITI than the R period (20–140 ms: *p* < 0.001), and longer replay epochs during the R period than the ITI (420–1100 ms, *p* = 0.007; 1200–3000 ms, *p* < 0.001; Fig. 4*B*).

The analysis was repeated after introducing the null category (N) for no replays. We introduced a threshold for rejecting the classifier outputs that were close to classification decision boundaries. We labeled those rejected classifier outputs as null. For measuring the threshold, we first extracted the probability distribution of the distance to the classification boundaries (*d*) obtained from the R and the ITI periods (Fig. 4*C*). The applied classifier was accurate 78% of the time. We then selected a conservative threshold (d* = 2.49) to reject 22% of outputs of the classifiers decoding the patterns during the retention period that were closest to the classification boundaries (they were the 22% that were most ambiguous). The same threshold rejected 94% of the decoded patterns during ITI period. We labeled these rejected time bins as N for null.

After applying the threshold, the overall number of replays of 170 ms representations (F, B, and C) was higher during the R period (5422, SD = 1061) than in the ITI (92, SD = 149; *p* < 0.001), and the number of Ns (rejected bins) was higher during the ITI (12,657, SD = 2961) than during the R period (3058, SD = 1669; *p* < 0.001). Furthermore, the decoded epochs were longer during the R period than during the ITI (in all four length bins, *p* < 0.001; Fig. 4*D*), meaning that the replayed stimuli persisted over a longer time during the R period. These results indicated that during the retention period one stimulus was PM. There was no significant interaction between stimulus category and order and the predominant stimuli (*F*_(4,52)_ = 0.603, *p* = 0.662) and no main effects of order (*F*_(2,26)_ = 0.747, *p* = 0.484) or stimulus category (*F*_(2,26)_ = 0.701, *p* = 0.505; Fig. 4*E*). At a group level, the length of replay epochs for the predominantly maintained category was shorter than 160 ms in 25% (SD = 11.2) of trials, between 160 and 400 ms in 18.7% (SD = 4.5) of trials, between 420 and 1100 ms in 15.1% (SD = 3.8) of trials, and larger than 1100 ms in 41.3% (SD = 14) of trials.

### No evidence for replay in sequential order

**Figure 5.**
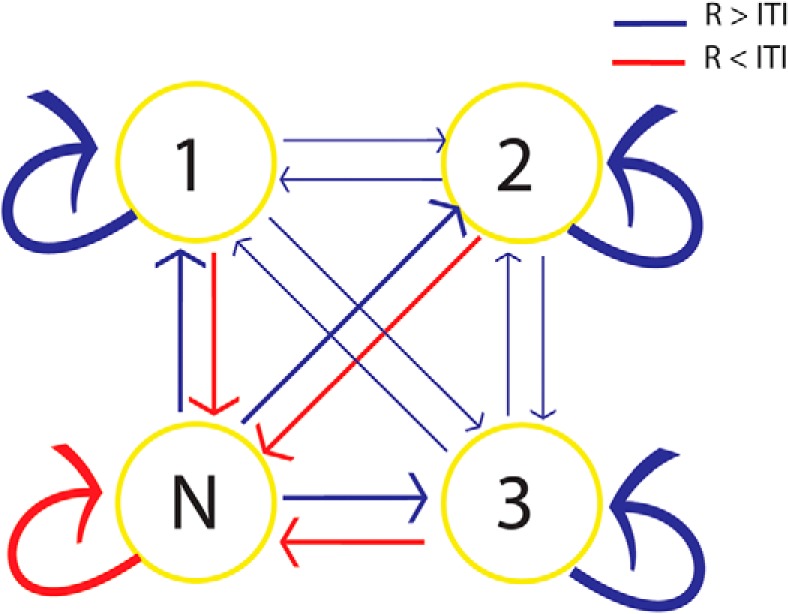
Difference in the averaged probability of state transition matrix is reflected by the thickness of the arrows. The probabilities of all transitions were different between the R periods and ITIs. Red arrows show the transitions that occurred more often during the ITI than the R period, and blue arrows show the opposite situation. There was no difference between the probabilities of forward (1-2-3) and backward (3-2-1) transitions.

The difference between the pattern of replay during the R period and the ITI was also detectable from the probability of replay of each stimulus at time bin *t* + 1 given the replay of a stimulus at time bin *t* (i.e., one-step, discrete-time Markov chain transition matrix between replayed states). If at time *t* a stimulus replays, most probably at time *t* + 1 the same stimulus will replay (averaged probability of transition was 56.32%). Probabilities of transitions to the same state and from N to each of the stimulus states were higher during the R period than during the ITI, and the probabilities of transitions from any state to N were lower during the R period than during the ITI. There was no difference between forward and backward transitions (Fig. 5).

### Enhanced memory recall for the dominantly replayed stimuli

We then examined the behavioral performance for replayed stimuli by fitting a linear mixed-effects model; that is, the length of (longest) consecutive replay of the probes in each trial as a fixed variable and the subject identity as a random variable. The results showed significant effects of length of replay on the performance for detail test (parameter estimate, 0.0001; *t*_(2232)_ = 2.578, *p* = 0.01) and on response time for the detail test (parameter estimate, −0.63175; *t*_(2232)_ = −2.115, *p* = 0.0345). The result was not significant for the performance of order test (parameter estimate, <0.0001; *t*_(2232)_ = 0.47757, *p* = 0.633) or the response time of the order test (parameter estimate, 0.39498; *t*_(2232)_ = 1.1955, *p* = 0.232).

**Figure 6.**
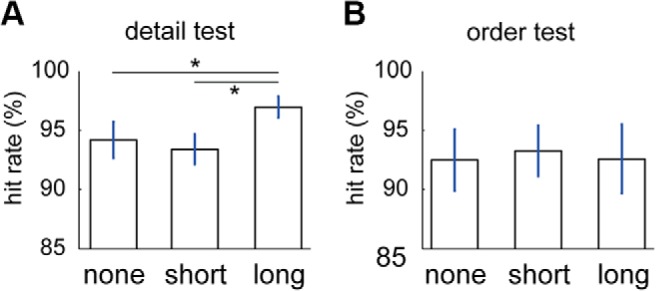
Effect of replay of 170 ms representation on WM performance. ***A***, ***B***, The detail test (***A***) and for the order test (***B***) show the hit rate (%) with respect to whether the stimuli were not replayed (none), were replayed for a short duration (shorter than 1100 ms), or replayed for a long duration (longer than 1100 ms). Error bars show the SEM. *p < 0.05.

We considered how long the longest replay epoch of the probe was during the preceding retention interval. We grouped the probes into the following three groups: those with no replay [detail test, 72.1 probes (SD = 9.9); order test, 73.6 probes (SD = 12.4)]; a short replay epoch [<1100 ms; Fig. 4, first three bars); detail test, 64.1 probes (SD = 16.8); order test, 64.4 probes (SD = 16.1)]; and a long replay epoch [>1100 ms; Fig. 4, last bar; detail test, 23.8 probes (SD = 9.7); order test, 21.9 probes (SD = 8.9)]. We also tested the behavioral responses according to how long the probe replayed during retention. The effect of the length of replay epoch predicted accuracy in the detail test (the first test the subjects performed after the retention period; *F*_(2,26)_ = 4.98, *p* = 0.015). The *post hoc* test showed that the hit rate was higher for the probes with long replay epochs than those with short replay epochs (*t*_(13)_ = 2.78, *p* = 0.016) or those not replayed (*t*_(13)_ = 2.85, *p* = 0.014; Fig. 6). We did not find any effect of replay on detail test response time (*F*_(2,26)_ = 1.89, *p* = 0.17), order test response time (*F*_(2,26)_ = 0.20, *p* = 0.82), or order test accuracy (*F*_(2,26)_ = 0.12, *p* = 0.89).

### Event-related activity during encoding predicts item replay

**Figure 7.**
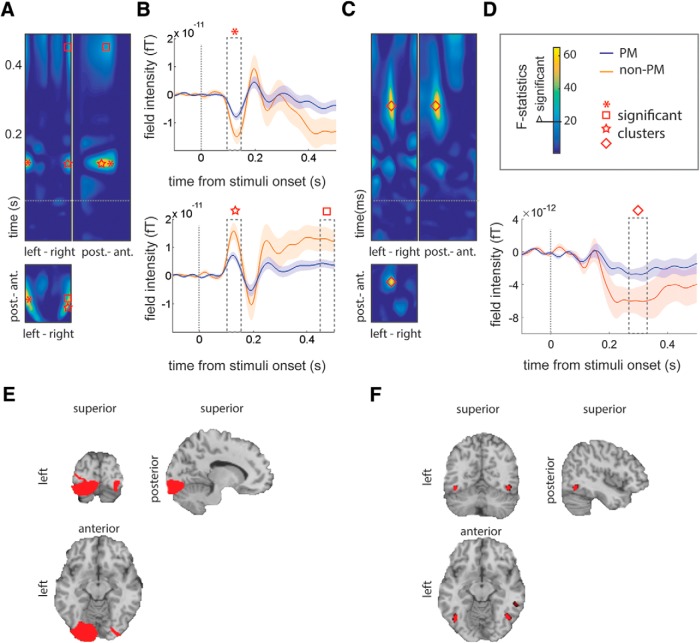
ERFs during encoding differentiate between PM stimuli in working memory and non-PM stimuli. ***A***, The plots graph the *F*-statistics in channel by time topography, focusing on the significant clusters at 125 ms from the stimuli onset. The bottom plot shows the channel by channel topography of the effect (*x*-axis is from left to right, and *y*-axis is from posterior to anterior). The top plots are channel by time. The *x*-axis on the left plot shows channels from left to right, and the *x*-axis on the right plot shows the channels from anterior to posterior. The peaks are highlighted with shapes in ***A*** to ***D***. ***B***, The top plot is for the effect that peaked at 125 ms (*p* < 0.001) in a left lateral channel, and the bottom plot is for the ERF effect at 453 ms (*p* = 0.008) in a right lateral channel. The plots show the ERF effects in the peak of significant clusters, which are highlighted by shapes (***A*** and ***B***). The dashed boxes show the timing of the effects. ***C***, The plots graph the *F*-statistics in channel by time (the same as in ***A***), focusing on the significant effect that peaked at 287 ms (*p* = 0.002). The effect is highlighted by a diamond shape in ***C*** and ***D***. ***D***, The plot shows the ERF effect at 287 ms from the stimuli onset in middle frontal channels. ***E***, The ERF effect at 125 ms (***A*** and ***B***) was source localized in the bilateral occipital cortex. ***F***, The ERF effect at 287 ms (***C*** and ***D***) was source localized in the posterior inferior temporal areas. ***A*** to ***D***, Dotted line shows the onset of the stimuli at encoding.

ERFs during encoding were examined as a function of which item was PM during the retention period. The ERFs were preprocessed in exactly the same way as the signal for pattern classification analysis and were low-pass filtered at 20 Hz. The results revealed that PM and non-PM stimuli during encoding evoked significantly different ERFs at right temporal channels (peaked at 125 ms; *F*_(2,26)_ = 44.14, *p* < 0.001, FWE corrected) and left temporal channels (peaked at 115 ms; *F*_(2,26)_ = 39.25, *p* < 0.001, FWE corrected; and later peaks at 453 ms; *F*_(2,26)_ = 23.06, *p* = 0.008; Fig. 7*A*,*B*), as well as at middle frontal channels (peaked at 287 ms; *F*_(2,26)_ = 32.49, *p* = 0.002, FWE corrected; Fig. 7*C*,*D*). The early ERF component (peaking at 125 ms) was source localized to the occipital temporal and the medial temporal cortices in both the left and right hemispheres (Fig. 7*E*). The difference was significant in left occipital (*F*_(1,13)_ = 36.51, *p* = 0.027, FWE corrected; Fig. 7*E*). The later ERF component, which peaked at 287 ms, was source localized to three brain regions, one on the left inferior temporal cortex (*F*_(1,13)_ = 21.85, *p* = 0.033, FWE corrected; Fig. 7*F*) and two on the right inferior temporal cortex (*F*_(1,13)_ = 20.44, *p* = 0.036, FWE corrected; and *F*_(1,13)_ = 19.03, *p* = 0.42, FWE corrected; Fig. 7*F*).

## Discussion

Using MEG, we decoded the working memory content while individuals maintained the sequence and the visual details of three distinct stimuli. Our results revealed that one of the three stimuli dominated the content of working memory. The predominantly maintained item benefited memory performance, akin to the behavioral effect of retaining an item on the focus of attention (Lepsien and Nobre, 2007; Lepsien et al., 2011; Gazzaley and Nobre, 2012; Tan et al., 2014). The item selected for preferential replay was not predicted by the identity or the sequence position (Fig. 4*E*). Instead, the predominantly maintained stimulus was selected based on the lowest amount attention related ERF amplitude during encoding (Fig. 7).

Our strict criterion for the existence of a sequential replay was the probability of sequential transitions in a discrete-time (one-step) Markov chain transition matrix (Fig. 5). Accordingly, we did not find directional replay; namely, any differences between the forward replay (1, 2, and then 3) or backward replay (3, 2, and then 1; Fig. 5). In addition to this strict criterion, we tested a direct prediction of the temporal coding model. The temporal coding model predicts that all three memoranda would be decoded with equal probability during maintenance. This criterion was also not fulfilled (Fig. 6). These null findings have to be interpreted with caution because the spatiotemporal resolution of our methodology may not be sensitive to sequential replay and direct intracranial recording may be required to provide further evidence for or against these models. Furthermore, sequential replay may be recruited with higher working memory load than that used in the current study (Heusser et al., 2016).

We observed that one stimulus dominated during the retention (Fig. 4). The identity of this stimulus varied from trial to trial. As noted, the category or the order of sequence did not determine what stimulus would replay (Fig. 4*E*). Instead, it was the amplitude of the ERFs at 125 ms from stimulus onset during encoding that predicted what stimulus would replay (Fig. 7). The early effect was source localized to left extrastriate cortex (Fig. 7), and this spatiotemporal pattern corresponds closely to the well known effect of attention to a visual stimulus during encoding (Heinze et al., 1990; Luck et al., 1990; Okazaki et al., 2008; Rutman et al., 2010). Attention to a visual stimulus elicits an enhanced event-related component in the occipital cortices (Hopf et al., 2000). Specifically, allocating attention to visual stimuli increases the magnitude of event-related EEG and MEG amplitude at ∼100 ms after the onset of visual stimulus relative to less attended stimuli (Hillyard and Anllo-Vento, 1998; Downing, 2000). Thus, stimuli that dominated replay during the retention interval were those that had received the least early attention allocation during encoding. This early reduced attention effect on the weakest encoded event was followed by a reduced amplitude event-related response at 287 ms that source localized to posterior inferior temporal regions. This indicates that the diminished early visual attention was followed by weaker representations in downstream visual areas.

Our findings are compatible with longstanding research on how attention can influence the content of working memory. Multiple items in working memory are not all in the same representational state during retention due to attention allocation (Zokaei et al., 2014; Myers et al., 2017). Rather, brain stimulation or experimental instructions to maintain a prompted stimulus (i.e., retro-cue procedure) manipulates the content of retention (Lewis-Peacock and Postle, 2012; Zokaei et al., 2014; Rose et al., 2016). Retro-cuing shifts the prompted stimulus into “the focus of attention.” In our experiment, we did not use retro-cues or brain simulation; instead, all three visual items were task relevant. This procedure allowed us to uncover an uninstructed prioritization of working memory content that was dependent on the degree of early attention.

Our observation that one item can dominate the maintenance period is compatible with recent neurophysiological data from the prefrontal cortex (PFC) of nonhuman primates. These effects of replay on behavior suggest that only the item in the focus of attention is actively replayed in working memory, while the representation of other stimuli are in an “active-silent” state (Sandberg et al., 2003; Stokes, 2015). The active-silent state is proposed to be a form of synaptic level retention where single-unit activity drops to baseline levels after an initial firing burst (Mongillo et al., 2008; Stokes, 2015; Lundqvist et al., 2016).

An intriguing question raised by our data is how the weakly encoded stimuli are prioritized for maintenance. Since prioritization was independent of sequence position, it could have occurred only after all three stimuli were encountered. A parsimonious scenario is that maintenance prioritization occurs at the beginning of the delay period (perhaps in the PFC; Lundqvist et al., 2016) and involves the retrieval of information. One possibility is that the prioritized stimulus required more search or retrieval effort during the delay. Such a process could have been supported by prefrontal mechanisms allowing monitoring (Barbey et al., 2013; Szczepanski and Knight, 2014) and inhibitory control (Knight et al., 1999; Barceló et al., 2000; Aron et al., 2004) reducing interference (LaRocque et al., 2014; Zokaei et al., 2014) from strongly encoded stimuli. This potential mechanism would compensate for capacity limitations of working memory (Luck and Vogel, 1997; Awh et al., 2006; Bays and Husain, 2008; Bays et al., 2009) and would be more resource effective by prioritizing the less privileged stimuli at encoding in the maintenance buffer. In essence, the subjects enhanced the replay of poorly attended stimuli to improve subsequent performance. Whether more strongly attended (higher-amplitude early ERFs) stimuli were encoded into and retrieved from long-term memory or whether they were in an active-silent state (Stokes, 2015; Lundqvist et al., 2016) remains an open question. Another option is that items were sequentially replayed but when the signal for the weakly attended item was amplified, this masked the decoding of other items.

In summary, we decoded the dynamic replay of the content of visual working memory with high temporal resolution using MEG. The results revealed that the representation of visual categorical information of the least attended stimuli during encoding was preferentially replayed during retention. These findings reveal that working memory maintenance intelligently prioritizes the weakest attended and encoded task-relevant stimuli, enhancing the fidelity of memory recall.

## Synthesis

Reviewing Editor: Bradley Postle, University of Wisconsin Decisions are customarily a result of the Reviewing Editor and the peer reviewers coming together and discussing their recommendations until a consensus is reached. When revisions are invited, a fact-based synthesis statement explaining their decision and outlining what is needed to prepare a revision will be listed below.

I've looked over your response to the Journal of Neuroscience reviews, and feel that, with one important exception, you have done a very thorough and appropriate job of responding to concerns both in your responses and in the revised manuscript that you've sent to eNeuro. The one area that still needs attention, I think, with with regard to your failure to find evidence for the phase-encoding model. Both of the Journal of Neuroscience reviews raise the point that you may be making stronger assertions about the 'disproving' of this model then can be supported by your data. In response, you argue that ‘If the ramifications of a model cannot be empirically observed than that model is useless. What is the point of a model in the absence of empirical support? Indeed, the temporal coding model makes, all electrophysiological intricacies aside, a very simple prediction: decoding shows equal probability for all to-be-retained items.’ Although I agree, in principle, with the rhetorical question that you pose, I nonetheless see several problems with the line of argumentation that follows. First, in the whole of the scientific literature, there are many instances of empirical support from this model, and so one could argue that this model isn't subject to your rhetorical argument. Second, it's not clear to me that the phase-encoding model makes the prediction that you make for it in as unqualified a manner as you portray it. For example, one could imaging that BOTH the phase-encoding model and your model are true: each to-be-remembered item is refreshed once per theta cycle, but the magnitude of the signal associated with each item is biased such that it's largest for the item that evoked the weakest response at encoding. If this were true, one could imagine that this bias plays out such that, with the decoding that you are using, you are much more likely to detect evidence for the favored item. My top-of-the-head story may or may not be more compelling than yours, but the point is that it's too strong to say that your data DEFINITIVELY REJECT the phase encoding model.

Here's what I propose: You've got a strong and novel finding just in terms of the delay-period bias that favors the most weekly encoded item. If you can reframe the paper such that it emphasizes this finding, while also reporting and interpreting your failures to find evidence for phase encoding (but not making strong claims about having rejected that model), then I'd be prepared to accept such a resubmission without sending it out for review. If, however, you're not willing to make this modification, then I'll need to send it out for review, just as I would a de novo submission.
